# Uncovering latent biological function associations through gene set embeddings

**DOI:** 10.1186/s12859-025-06100-9

**Published:** 2025-03-24

**Authors:** Yuhang Huang, Fan Zhong, Lei Liu

**Affiliations:** 1 Institutes of Biomedical Sciences, Fudan University, 131 Dongan Road, Shanghai, 200032 China; 2 Intelligent Medicine Institute, Fudan University, Shanghai, 200032 China; 3 Shanghai Institute of Infectious Disease and Biosecurity, Fudan University, Shanghai, 200032 China; 4 Shanghai Institute of Stem Cell Research and Clinical Translation, Shanghai, 200120 China

**Keywords:** Biological network analysis, MSigDB, Gene-term associations, Cross-species analysis, Network embedding

## Abstract

**Background:**

The complexity of biological systems has increasingly been unraveled through computational methods, with biological network analysis now focusing on the construction and exploration of well-defined interaction networks. Traditional graph-theoretical approaches have been instrumental in mapping key biological processes using high-confidence interaction data. However, these methods often struggle with incomplete or/and heterogeneous datasets. In this study, we extend beyond conventional bipartite models by integrating attribute-driven knowledge from the Molecular Signatures Database (MSigDB) using the node2vec algorithm.

**Results:**

Our approach explores unsupervised biological relationships and uncovers potential associations between genes and biological terms through network connectivity analysis. By embedding both human and mouse data into a shared vector space, we validate our findings cross-species, further strengthening the robustness of our method.

**Conclusions:**

This integrative framework reveals both expected and novel biological insights, offering a comprehensive perspective that complements traditional biological network analysis and paves the way for deeper understanding of complex biological processes and diseases.

**Supplementary Information:**

The online version contains supplementary material available at 10.1186/s12859-025-06100-9.

## Introduction

The intricate architecture of biological systems has been progressively demystified through advanced computational methodologies. Currently, biological network analysis predominantly focuses on constructing and interrogating well-defined interaction networks. These approaches often employ graph-theoretical methods to identify key nodes and critical pathways, utilizing high-confidence interaction data to map out precise biological processes. Databases such as KEGG [1] and STRING [2] are commonly used.

Initially, researchers employed network-based methods primarily to analyze biological data [3]. Subsequent advancements have profoundly deepened our understanding of biological networks through unsupervised analysis. Jeong et al*.* [4] unveiled complex structures within metabolic networks, Barabási and Oltvai [5] elucidated the functional organization of cellular networks, and Stuart et al*.* [6] were pioneers in identifying conserved gene modules across species. Cumulatively, these seminal studies underscore the evolution and significant impact of network analysis in bioinformatics. Within the past decades, researchers have been applying machine learning on graphs and utilizing various methods to represent [7]: from shallow embedding approaches like DeepWalk [8], node2vec [9], and HARP [10], to methods based on deep learning like neighborhood autoencoder methods DNGR [11], SDNE [12], and convolutional one like GraphSAGE [13]. Among these methods, node2vec stands out due to its simplicity and strong interpretability in an unsupervised learning context.

In the field of computational biology, graph representation learning has gained popularity in the analysis of biological relationships [14, 15]. Over the past three years, numerous studies have reviewed and summarized its applications in the biomedical domain [16]. Most research has primarily focused on protein-drug associations and uncovering potential relationships among cell types or diseases through semi-supervised or unsupervised learning approaches. In particular, gene-expression matrices are often transformed into topological forms for analysis. For instance, the GCN-MF [17] developed by Han et al*.* effectively captures nonlinear associations between genes and diseases using semi-supervised learning. Subsequently, they introduced VGE [18], which utilizes an autoencoder to generate potential distributions in a novel semantic embedding space, improving the prediction of new disease-gene associations. Similarly, this approach has been applied to analyzing cell-type associations by leveraging transcriptomic data with robust models such as DREAMIT [19], Cellograph [20], and DEGAS [21], all of which employ graph neural networks (GNN). These studies aim to enhance model discriminative capabilities. In our research, we adopt the core principle of embedding while utilizing a simpler and more interpretable GNN to investigate these relationships.

**Fig. 1:**
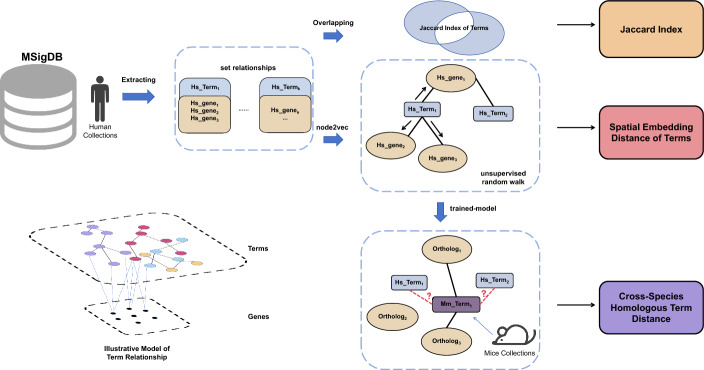
Network-based framework for exploring complex relationships between biological terms

Building on advancements in graph-based representation learning, this study extends traditional bipartite models by incorporating attribute-driven knowledge database, Molecular Signatures Database (MSigDB) v2023.2 [22]. Traditional biological network analyses, while effective with specific, well-curated datasets, often falter when faced with incomplete or ambiguous data from diverse sources [23]. To bridge this gap, we introduce a novel network-based framework designed to explore complex relationships within biological terms (Fig. 1). Rather than relying solely on annotated data, our approach uncovers potential gene associations with functional terms (e.g., pathways, functions) through network connectivity. By analyzing network structure and topology, we aim to reveal hidden patterns and novel associations, offering deeper insights, particularly in exploratory studies where direct evidence is limited or mechanisms are unclear. This integrative approach enhances traditional data mining by offering a more comprehensive perspective.

Using the *Jaccard index* and node2vec-based distance, we analyzed and embedded human and mouse datasets into a shared vector space. Homologous gene alignment across species highlighted consistent relational patterns, reinforcing the robustness of the findings. This figure focuses on the connections between terms, with the abstract representation of these relationships shown in the diagram at the lower left.

## Method

### Data integration

The dataset incorporates a comprehensive set of terms that encapsulate a wide array of biological phenomena, we have opted to focus on a single species to mitigate these potential confounders and to ensure the integrity of the analysis. Within this scope, we define two essential sets: the set of **genes**, denoted as $G=\{{g}_{1},{g}_{2},...,{g}_{m}\}$, and the set of **terms**, denoted as $T=\{{t}_{1}{,t}_{2},...,{t}_{n}\}$. Data for these sets were sourced from the MSigDB (https://www.gsea-msigdb.org/gsea/msigdb). To bridge species-specific differences in gene nomenclature, we utilized the R package biomaRt [24], which facilitated the conversion of murine genes to their human orthologs, ensuring cross-species comparability in our analysis.

Following data preprocessing, we construct an index dictionary to facilitate the creation of a PyG [25] (PyTorch Geometric) data object. This entails defining two mappings: one from genes to indices, termed gene_to_index: $G\to \{\text{0,1},...,m-1\}$, and another from terms to indices, termed term_to_index: $T\to \{m,m+1,...,m+n-1\}$.

We then generate the list of edges by adding an edge for each gene-term pair (*g,t*) to form the edge set $E=\{(\text{gene}\_\text{to}\_\text{index}(g),\text{term}\_\text{to}\_\text{index}(t)\}$*.* The PyG data object, represented as $Graph=(V,E)$, encompasses the node set $V=G\cup T$*,* and the edge set *E*, where the number of nodes $\left|V\right|=m+n$.

### Reliability score

Our analysis revealed that across 10^8^ pairwise comparisons, both the *Jaccard index* and the distances in the node2vec embeddings exhibited distributions with extreme values. To assess significant biological associations, we normalized both metrics and selected representative pairs at distance thresholds of 0.1, 0.3, 0.5, 0.7, and 1 for further manual evaluation and literature-based validation. For each threshold, 10 pairs were analyzed in-depth to determine their biological relevance. To enhance the rigor of biological relevance assessment, we introduced a reliability score (*RS*) ranging from 0 to 5 (Table 1), reflecting levels of confidence from speculative associations to those strongly supported by extensive literature (Supplementary Material 1).

**Table 1:** The criteria for *RS* assignment

Reliability score	Descriptions
5 (Strong evidence)	The association is strongly supported by multiple independent studies and extensive literature evidence, showing clear biological relevance
4 (Reported, with inference)	The association has been reported in the literature, but its biological relevance requires some level of inference or additional interpretation
3 (Weak association)	The association has limited evidence in the literature, often mentioned in less direct contexts or without detailed mechanistic support
2 (Speculative)	The association lacks direct literature evidence but is suggested by indirect data or weak connections
1 (Minimal overlap)	The association is based on minimal overlap between gene sets or datasets, suggesting a tenuous link that is mostly speculative
0 (No association)	The association is deemed biologically implausible, such as involving genes located on different chromosomes without known interactions

### Distance calculation methodology

We utilized the node2vec algorithm to embed nodes within our graph-structured data, capturing their contextual relationships. The node2vec algorithm learns these embeddings by performing biased random walks on the graph, balancing between breadth-first search (BFS) and depth-first search (DFS) through two parameters: return parameter $p$, and in–out parameter $q$.

The random walks are controlled by the following transition probability formula:

$$P({c}_{i}=x \mid {c}_{i-1}=v) = \begin{cases} \frac{1}{p} & \text{if } d(v,x)=0 \\ 1 & \text{if } d(v,x)=1 \\ \frac{1}{q} & \text{if } d(v,x)=2 \end{cases}$$

where *c*_*i*_ is the candidate note at step *i*, ${c}_{i-1}=v$ is the previous node in the walk, $d(v,x)$ is the shortest path distance between nodes $v$ and $x$.

The *Jaccard index* measures the overlap between two sets, and in our context, it represents the similarity between terms based on the overlap of their corresponding gene sets. Consider two terms, ${T}_{\text{A}}$ and ${T}_{\text{B}}$, each associated with a set of genes based on their adjacency in the graph structure.

The *Jaccard index* between these two terms is calculated using the formula:

$$\text{J}({T}_{\text{A}},{T}_{\text{B}})=\frac{|{T}_{\text{A}}\cap {T}_{\text{B}}|}{|{T}_{A}\cup {T}_{\text{B}}|}$$

### Node2vec training and hyperparameter optimization

To uncover the underlying patterns within our data, which involve complex many-to-many relationships without precise numerical values, we have adopted an unsupervised learning approach. Central to this approach is the node2vec algorithm, as implemented in the torch_geometric library, which we integrated into a machine learning pipeline. This pipeline enables the sequential execution of node embeddings and subsequent clustering.

Clustering is essential in unsupervised learning as it allows us to organize data points into groups based on their inherent similarities, without relying on pre-labeled classes. The hyperparameter tuning for the node2vec model involved varying several parameters (Table 2).

**Table 2:** Hyperparameter search for node2vec

Parameter	Values tested
Embedding dimensions	32, 64
Walk length	10, 20, 30
Return parameter (*p*)	0.25, 0.5, 1, 2, 4
In–out parameter (*q*)	0.25, 0.5, 1, 2, 4
Context size	10, 20
Negative samples	1, 5, 10
Number of walks per node	10, 20, 30

Following the optimization of hyperparameters, we applied *k*-means clustering to evaluate the quality of the embeddings. The cluster labels assigned by *k*-means were used to calculate two internal clustering validation metrics, which assess the coherence and separation of the clusters.

The *Silhouette score* [26] ranges from −1 to 1, with higher values indicating better clustering performance. It is defined as:

$$S=\frac{1}{n}\sum_{i=1}^{n}\frac{{b}_{i}-{a}_{i}}{\text{max}({a}_{i},{b}_{i})}$$

where ${a}_{i}$ is the average distance between the $i$-th data point and other points in the same cluster, ${b}_{i}$ is the minimum average distance from the $i$-th data point to the points in a different cluster, $n$ is the total number of data points.

The *Davies-Bouldin score* [27] measures the average similarity between clusters, with a lower values indicating better clustering performance. It is calculated by:

$$DB=\frac{1}{k}\sum_{i}^{k}\underset{j\ne i}{\text{max}}(\frac{{\sigma }_{i}+{\sigma }_{j}}{d({c}_{i},{c}_{j})})$$

where ${\sigma }_{i}$ is the average distance of all points in cluster $i$ to the cluster centroid, $d({c}_{i},{c}_{j})$ is the distance between centroids of clusters $i$ and $j$, while $k$ is the number of clusters.

The 64-dimensional space is more likely to exhibit biological credibility, as evidenced by supervised analyses where embeddings of this dimensionality achieve higher Micro-F1 and Macro-F1 scores across benchmark datasets [9]. Furthermore, we observed that the 32-dimensional embeddings produce less credible results within the top 5%. Ultimately, we utilized the 64-dimensional embeddings for the subsequent statements of node2vec-based distances.

The model was trained on a server equipped with an NVIDIA GeForce RTX 3080 Ti GPU, using CUDA for parallel computations. The server ran Ubuntu 20.04 LTS and had 256 GB of RAM. We used Python 3.8 and the torch_geometric library for our implementation. The hyperparameter search was conducted over the course of one week, with each parameter combination undergoing 40 epochs of training. The duration of training for each parameter set varied depending on the specific parameters chosen, with some combinations taking more time to complete.

## Results

### Interpreting biological relevance of *Jaccard Index*

**Fig. 2:**
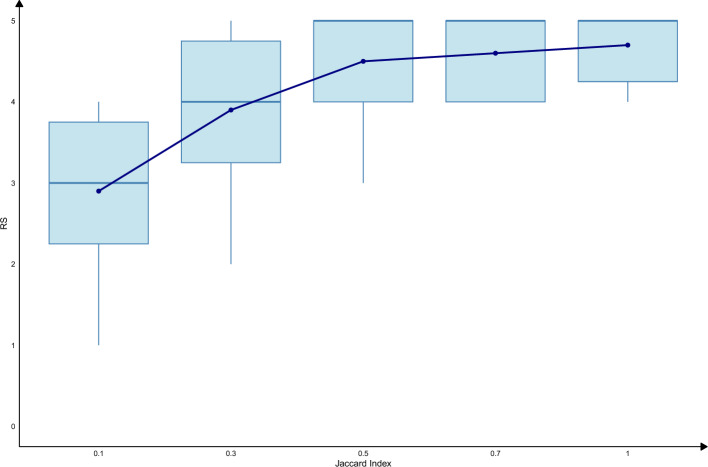
Relationship between the *Jaccard index* and *RS*

A higher *Jaccard index* generally signifies a substantial overlap between gene sets, indicating a robust biological connection. However, a lower *Jaccard index* should not be overlooked, particularly when the gene sets involved contain fewer genes, where even modest overlap might indicate meaningful associations. To refine the analysis, we applied a hypergeometric distribution test with a *p* value threshold of 0.05 to assess the significance of shared gene counts. From the analysis, it is evident that higher *Jaccard index* are generally associated with higher *RS* (Fig. 2), reflecting a trend that aligns with our expectations.

This box plot illustrates the distribution of *RS* across different *Jaccard index* values, highlighting their correlation. The *Jaccard index* quantifies the similarity between gene sets of biological terms, calculated as the size of the intersection divided by the size of the union of the gene sets. Higher values indicate greater overlap and stronger potential associations. *RS* represents the biological relevance of term pairs, ranging from 0 to 5. Each box represents the interquartile range (IQR), with the central line showing the median *RS* for term pairs at each *Jaccard index* level. Points outside the whiskers represent term pairs with exceptional RS values, indicating either unexpectedly high or low biological relevance given the *Jaccard index*. Higher *Jaccard index* values (e.g., 0.7–1) are generally associated with higher median *RS*, reflecting a trend that aligns with expectations of stronger biological relevance. Lower *Jaccard index* values (e.g., 0.1–0.3) exhibit greater variance in *RS*, suggesting these pairs may include a mix of weakly and potentially novel associations.

For example, the association between GOBP_UBIQUITIN_DEPENDENT_GLYCOPROTEIN_ERAD_PATHWAY and GOMF_MANNOSYL_OLIGOSACCHARIDE_1_2_ALPHA_MANNOSIDASE_ACTIVITY (with a *Jaccard index* of 1) is biologically intuitive. Although this connection is not explicitly indicated in MSigDB, these terms describe closely related processes. The ubiquitin-dependent glycoprotein ERAD pathway is responsible for recognizing and degrading misfolded glycoproteins in the endoplasmic reticulum (ER) via the ER-associated degradation (ERAD) system [28]. During this process, mannosyl oligosaccharide 1,2-alpha mannosidase enzymes trim misfolded glycoproteins, preparing them for ubiquitination and degradation. The inclusion of this enzyme activity within the pathway underscores the functional cohesion, highlighting the expected biological relationship between the two functional terms.

**Fig. 3:**
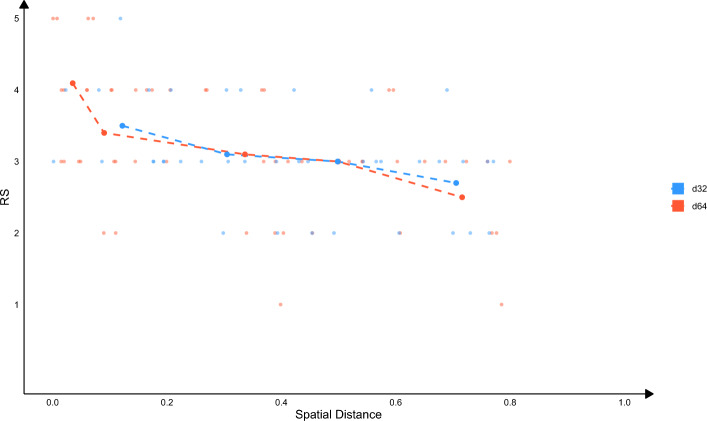
Linear correlation of *RS* and node2vec distances in 64- and 32-dimensions

As the *Jaccard index* decrease, a turning point emerges around 0.5, where *RS* typically ranges between 3 and 4 (Fig. 3). Although direct literature support becomes sparse, these associations remain biologically plausible. A notable example is the interaction between FU_INTERACT_WITH_ALKBH8 and GOCC_CHAPERONIN_CONTAINING_T_COMPLEX (CCT). While no explicit link has been documented between the CCT complex and ALKBH8, both share a key gene, *TCP1*, suggesting a potential functional connection. CCT, a type II molecular chaperone in eukaryotes, plays a pivotal role in folding nascent proteins, particularly during T-cell activation and in response to external stressors. Its critical function in maintaining protein homeostasis means that CCT deficiency can lead to immune dysfunction and impaired stress response. Conversely, ALKBH8, an enzyme responsible for tRNA modification, ensures translation accuracy and regulates oxidative stress by modifying the wobble position (e.g., mcm5U). Loss of ALKBH8 can result in neurological disorders and dysregulated oxidative stress responses [29]. In a stress environment, accurate protein folding and translation are crucial. Although CCT and ALKBH8 operate at different molecular levels—CCT ensuring proper protein folding and ALKBH8 ensuring translation fidelity—they converge on the broader roles of stress management and protein quality control. Deficiencies in tRNA modification, such as those caused by ALKBH8 dysfunction, could increase the burden of misfolded proteins, thereby heightening the reliance on the CCT complex for correction [30]. Thus, despite the moderate *Jaccard index*, the inferred connection suggests a meaningful biological interplay between these components.

This figure illustrates the linear correlation between *RS* and node2vec distances in both 64-dimensional and 32-dimensional vector spaces. The scatter plot displays individual term pairs, with *RS* (0–5) plotted against node2vec distances. A stronger negative correlation is observed in the 64-dimensional embeddings, where shorter distances (e.g., < 0.1) consistently correspond to higher *RS*, indicating a tighter coupling of spatial proximity and biological relevance. This suggests that the 64-dimensional embedding captures nuanced relational patterns more effectively than the 32-dimensional embedding, which exhibits greater variability at similar distances. The observed trend highlights node2vec’s capacity to encode biologically meaningful relationships, particularly at finer scales, where embedding quality directly influences interpretability and downstream analyses.

### Comparative analysis of node2vec and other embedding methods in network analysis

Addressing the challenges of incomplete and heterogeneous datasets that limit traditional graph-theoretical methods, our study explores the utility of node2vec and compares it with other graph embedding techniques, including FastMap [31], GraphFactorizations, LINE [32], and RandomProjection. This study highlights the strengths and limitations of node2vec in specific contexts, offering a balanced view of its performance.

While traditional graph-theoretical methods often struggle with missing nodes or edges, node2vec leverages biased random walks to enrich embeddings with meaningful context, allowing for the discovery of potential associations between indirectly related nodes. This makes node2vec particularly useful for sparse graphs. Through the combination of MSigDB with node embeddings, we successfully bridge diverse data types, linking genes and biological or clinical terms within a unified framework. Node2vec proved effective in incorporating such heterogeneous data, providing biologically interpretable insights. Node2vec showed a tendency to focus on associations with moderate *RS* (3–4), indicating its strength in uncovering potential relationships that may not be immediately evident but are biologically relevant upon closer examination. By contrast, methods like LINE and RandomProjection were better at preserving high-*RS* relationships between semantically similar terms.

We evaluated these embedding methods by manually reviewing the top 100 pairwise term distances and assessing their relevance using *RS* (Supplementary Material 2). LINE and RandomProjection achieved higher average *RS*, primarily excelling in identifying semantically close and well-documented term pairs. However, these methods demonstrated limited flexibility in capturing the deeper structure and complex relationships within the network, often prioritizing relationships that are already well-established in databases. In contrast, node2vec provided a more versatile approach, uncovering a broader range of connections that are less immediately apparent but biologically interpretable upon closer examination. While its average *RS* was lower, node2vec demonstrated strengths in capturing potential associations beyond semantically similar terms. Notably, even though semantically close terms appeared less frequently in node2vec’s top 100 pairs, they were consistently well-represented across the entire network, showcasing the method’s robustness in identifying both obvious and latent relationships. FastMap performed poorly, failing to effectively capture meaningful relationships. While GraphFactorizations achieved relatively high *RS*, the method tended to generate locally optimized clusters dominated by specific types of terms (e.g., miRNA-related terms), limiting its utility in broader analyses.

### Identifying novel biological associations through node2vec-based distances

**Fig. 4:**
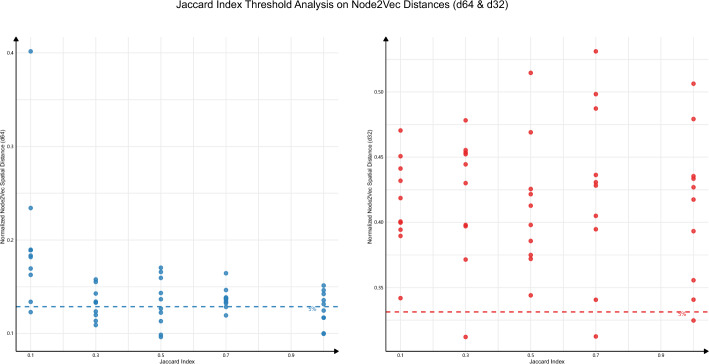
Correlation between *Jaccard Index* and node2vec distances in 64- and 32-dimensional spaces

Compared to traditional methods like the *Jaccard index*, which resemble differential gene analysis, node2vec results tend to have lower *RS* based on literature surveys. However, they hold greater research value, as node2vec-derived spatial proximity often correlates with phenotypic synergy. Additionally, comparisons between the *Jaccard index* and node2vec spatial distances reveal that the 64-dimensional embeddings (d64) include a higher proportion of the closest distances (top 5%), indicating higher reliability. Furthermore, the d64 scores consistently surpass that of the 32-dimensional embeddings (d32) in terms of reliability (Fig. 4), underscoring its superior performance in capturing meaningful biological relationships. We specifically focused on capturing those from the top 5% of the d64 distances. To further elucidate the relationships among terms, we developed a minimum spanning tree (MST, Supplementary Material 3) [33] and an interactive web interface.

This figure depicts the correlation between the *Jaccard index* and node2vec-calculated spatial distances in 64-dimensional (left) and 32-dimensional (right) embedding spaces. Each plot visualizes the distribution of term pairs based on their *Jaccard index* and corresponding node2vec distance, with normalized distance values ranging from 0 to 0.5 for d64 and 0.3 to 0.5 for d32. The dashed lines represent the top 5% threshold values, indicating d64 distance = 0.13 and d32 distance = 0.33, respectively. Term pairs below these thresholds are considered highly similar in the vector space. Despite a generally weak global correlation between the *Jaccard index* and node2vec distances, a clear pattern emerges in the 64-dimensional embedding: lower node2vec distances are associated with higher *Jaccard index*, suggesting that the spatial proximity in this embedding space reflects meaningful biological overlap. In contrast, the 32-dimensional embedding shows greater variability, with term pairs often scattered across a broader range of distances even at high *Jaccard index*. This observation shows that higher-dimensional embeddings better capture fine-grained biological relationships, particularly when evaluating term pairs with substantial gene overlap.

Our analysis reveals that a low *Jaccard index* does not invariably indicate a lack of functional or biological relevance, as several key genes across two distinct terms occupy crucial roles and upstream positions within the pathway. For instance, we observed a *Jaccard index* of merely 0.083 between the GNF2_PRDX2 gene set from the GNF2 expression atlas and the HP_ANISOCYTOSIS gene set. Despite the minimal overlap, the node2vec spatial distance between these terms’ ranks within the top 5%, hinting at a potential biological connection. This link may be driven by oxidative stress mechanisms, as PRDX2, an antioxidant enzyme, plays a pivotal role in regulating cellular redox balance. Oxidative stress is a key factor in various hematological disorders, and its modulation could influence red blood cell (RBC) morphology, specifically in conditions like anisocytosis, which is characterized by variations in RBC size. Altered PRDX2 expression has been associated with hematological conditions such as anemia, which frequently presents with anisocytosis. Specifically, aberrations in PRDX2 potentially influences cellular proliferation and contributes to various carcinomas. We hypothesize that PRDX2 may regulate RBC morphology by mitigating oxidative damage, thereby establishing a mechanistic link to anisocytosis. This potential connection between PRDX2 and RBC size variability could pave the way for further research into its role in hematological disorders, particularly those involving oxidative stress [34]. Despite the common clinical treatments for anisocytosis focusing on symptom management, such as vitamin B12 supplementation or blood transfusions, our findings suggest that targeting PRDX2-related pathways could offer a novel therapeutic perspective. Future experiments could include targeted gene editing techniques to modulate PRDX2 expression in model organisms or cell lines, observing resultant changes in RBC morphology and proliferation rates.

Both KEGG_MEDICUS_REFERENCE_TYROSINE_DEGRADATION and WP_TYROSINE_METABOLISM_AND_RELATED_DISORDERS are pathways associated with tyrosine metabolism and degradation. The former emphasizes the overall metabolic process, while the latter focuses on the pathological mechanisms resulting from metabolic disorders. Although these pathways share an identical gene set, their spatial distances in the node2vec analysis rank among the closest 5%, reflecting a stronger similarity in the embedding space. This suggests a tight biological network connection between these pathways and hints at potential clinical phenotypic commonalities.

In contrast, despite having a *Jaccard index* close to 1, KEGG_MEDICUS_REFERENCE_TRANSPORT_OF_CALCIUM and GOMF_GLUTAMATE_GATED_CALCIUM_ION_CHANNEL_ACTIVITY exhibit a noticeably larger spatial distance in the node2vec analysis and possess a *RS* that is four points lower than the others. The difference between these two terms lies in their perspective on the same activity; however, the former encompasses three fewer genes (*GRIA1*, *GRIA3*, *GRIK1*) than the latter. This highlights node2vec’s ability to distinguish between pathways based on their functional roles and pathological relevance, rather than being solely driven by gene set overlap. The KEGG pathway describes the broad physiological process of calcium ion transport across membranes, involving various cell types and tissues. In comparison, the GO molecular function category is more specific, focusing on the activity of glutamate-gated calcium ion channels within the nervous system, a more narrowly defined molecular mechanism. Consequently, node2vec separates the two terms in the biological network, reflecting differences in their functional and contextual roles despite their identical gene sets. This demonstrates node2vec’s capacity to capture topological distinctions in the network, offering a more nuanced understanding of functional relationships beyond simple gene overlap.

### Applying clinical insights to uncover complex biological relationships with node2vec-based distances

Building on our strategy involving node2vec-based distances, such as the insightful association between PRDX2 and anisocytosis, we explore more practical scenarios to deepen our understanding of complex biological processes and diseases. By harnessing clinically relevant term pairs, this approach aids in uncovering subtle yet significant biological interactions that may be overlooked by conventional methods.

In clinical research on immune diseases resulting from nephron developmental defects, we focused on the term HP_ABNORMAL_NEPHRON_MORPHOLOGY to identify critical genes implicated in these processes. Through *Jaccard index* and node2vec-based distance calculations, we expanded our analysis to related terms, such as HP_ABNORMAL_URINE_PROTEIN_LEVEL and HP_SKIN_RASH, which align with observed clinical manifestations and fall within the predefined screening criteria. These analyses have pinpointed key genes like *MEFV*, *IRF5*, *TNFAIP3*, and *PTPN22*, which are central to immune regulation and renal pathology. By narrowing the search space for candidate genes, our approach accelerates the discovery of up-downstream relationships in complex biological systems.

For instance, our term-driven analysis highlights *IRF5* and *STAT4* as pivotal genes linking proteinuria and skin rash in lupus nephritis (LN). These findings align with literature evidence [35], where these genes are shown to mediate interferon signaling and inflammatory responses. Moreover, pharmacological exploration, such as hydroxychloroquine (HCQ) modulation of these pathways, reveals dual effects [36]. Literature suggests that while HCQ improves renal inflammation and survival in LN, it may disrupt proximal tubular epithelial cell autophagy and accelerate cellular senescence, exacerbating susceptibility to acute kidney injury [37]. This demonstrates the complex interplay between genetic pathways and therapeutic interventions, reinforcing the importance of integrating term analysis with biological validation.

By combining term-based relationships and documented experimental evidence, our methodology offers a robust framework to rapidly identify and interpret key genetic pathways. It supports the generation of actionable hypotheses for experimental validation and enhances understanding of the molecular mechanisms underlying complex diseases.

### Cross-species discovery through node2vec embedding reveals shared biological insights

Due to ethical constraints prohibiting functional perturbation experiments within human subjects, alongside the limitations of cell line experiments that diverge from the true in vivo environment, a substantial amount of research is conducted within murine models. The central aim of these animal experiments is to bridge murine data with human disease and physiological mechanisms, rendering cross-species association studies indispensable.

We extended our analysis to include cross-species validation, utilizing node2vec unsupervised embeddings at 64 dimensions. By embedding both human and mouse gene datasets into a shared vector space, we aimed to identify biologically relevant patterns that are conserved across species. Specifically, we converted mouse genes to their homologous human counterparts and embedded the mice collections based on the pre-trained human model.

To ensure cross-species comparability, we mapped 41,662 mouse genes and 42,416 human genes, identifying 17,044 homologous gene pairs. While the homologous mouse genes represent less than half of the original set, the connections between homologous mouse genes and human terms increased four folds compared to their original connections to mouse terms. Importantly, both homologous genes, mouse terms, and human terms were embedded into the same shared space, facilitating the identification of conserved relationships. In our analysis, we prioritized biologically meaningful mouse terms (Table 3) and focused on examining their relationships with the nearest human terms. Following this, we searched for the nearest human term-related nodes corresponding to the embedded terms in mouse. This approach allowed us to discover and strengthen our findings across species (Supplementary Material 1).

**Table 3:** Key cross-species associations between mouse ageing data and human disease-related terms

Mice term	Human term	Biological interpretation
TABULA_MURIS_SENIS_LUNG_B_CELL_AGEING	CHARAFE_BREAST_CANCER_LUMINAL_VS_MESENCHYMAL_UP	Links ageing lung B cells with mesenchymal transitions in breast cancer progression
LIVER_T_CELL_INFLAMMATORY	HCC_TUMOR_MICROENVIRONMENT_UP	Suggests inflammation-driven tumorigenesis mechanisms in the liver
SPLEEN_MARGINAL_ZONE_B_CELL_AGEING	IMMUNE_CELL_FUNCTION_IN_BREAST_CANCER	Indicates possible changes in immune surveillance impacting tumor development

The observed connection between mice_TABULA_MURIS_SENIS_LUNG_B_CELL_AGEING and human_CHARAFE_BREAST_CANCER_LUMINAL_VS_MESENCHYMAL_UP suggests potential links between ageing lung B cells and distinct cellular states in breast cancer [38]. The mesenchymal transition in cancer is frequently associated with immune responses and cellular ageing, which might explain this correlation. Ageing B cells, particularly in tissues like the lung, may undergo significant functional changes, such as a reduction in antigen-specific responses or a shift towards pro-inflammatory profiles. These changes are characteristic of immunosenescence, which is known to both impair immune surveillance and enhance a pro-tumorigenic environment. Chronic inflammation, often seen with ageing immune cells, creates a microenvironment conducive to cancer progression through the secretion of inflammatory cytokines and growth factors that support tumor cell proliferation, survival, and metastasis. This is especially relevant to the mesenchymal transition in cancer, as inflammatory signals are critical drivers of epithelial-mesenchymal transition (EMT), facilitating cancer cell invasion and dissemination.

## Discussion

In this study, we utilized node2vec, comparing to straight forward set-overlapping methods, to explore complex biological relationships within diverse datasets. The key strength of this approach lies in its ability to embed terms from various datasets into a shared vector space, enabling researchers to discover relationships between similar terms across different datasets. This provides a flexible tool for uncovering hidden patterns, particularly in contexts where data is heterogeneous and often incomplete.

However, one of the limitations of this work is the reliance on pre-existing datasets, which introduces a level of uncertainty. Our term embeddings are drawn from various sources, each with varying levels of curation and confidence, and we have not assigned explicit weights to these terms yet. This results in a broad, unsupervised analysis where both input and output lack precise control. While this introduces potential noise and ambiguity, it also allows for serendipitous discoveries, as seen in some of the unexpected relationships we uncovered. These findings suggest that even in a loosely structured exploration, novel biological insights can emerge, particularly when the direct evidence is limited or the underlying mechanisms are not well understood. In addition, MSigDB was selected over other databases such as KEGG or Reactome due to its broad aspects of gene sets (terms) and inclusion of computationally derived gene clusters. Notably, MSigDB also integrates information from databases like Reactome and KEGG, providing a more comprehensive resource for general-purpose analyses. While Reactome and KEGG are highly curated and particularly well-suited for pathway-specific research or studies targeting detailed mechanistic insights, they are less versatile for exploratory or large-scale analyses involving heterogeneous datasets.

The node2vec algorithm itself alleviates some of the concerns mentioned above. Its simplicity and ease of parameter tuning make it well-suited for exploratory analyses. Additionally, its unsupervised nature is particularly advantageous when working with heterogeneous and incomplete data, which is common in biological research. This flexibility allows us to capture potential relationships that more rigid, supervised methods might overlook.

Despite these strengths, it is important to acknowledge that our study primarily serves as a proof-of-concept. The current framework demonstrates the potential of node2vec in biological research, but more concrete case studies with well-curated datasets are necessary to fully exploit its capabilities. The quality and scope of the analysis are ultimately constrained by the completeness and curation standards of the underlying data. To address this, future studies could incorporate complementary resources, to enhance the granularity and reliability of analyses, particularly in pathway-specific or clinical contexts. Furthermore, while node2vec offers simplicity and flexibility, its unsupervised nature limits its applicability in more targeted settings. Future work should therefore focus on integrating experimental validation and leveraging more sophisticated models, such as GNN, which may provide greater precision and sensitivity when tailored to specific research questions. These advancements would enable the transition of our framework from an exploratory tool to a robust platform for targeted biological discovery and clinical application.

## Supplementary Information

Additional file 1.

Additional file 2.

Additional file 3.

## Data Availability

The code utilized in this study is available on GitHub at https://github.com/YuHang-aw/MsigDB_mining.git (MIT license). The repository is licensed under the MIT license, facilitating reproducibility and supporting further research. A user-friendly Excel file detailing the relationships between term pairs is available in the repository and supplementary data, including values such as the Jaccard index, 64-dimensional distances (d64), and 32-dimensional distances (d32), which were obtained using the node2vec method. Additionally, the file includes comparisons of these distances with those generated by other embedding methods, along with evaluations of closely related term pairs. Raw MSigDB data can be downloaded from https://www.gsea-msigdb.org/gsea/msigdb/human/collections.jsp#H.
